# Combining RNA interference and kinase inhibitors against cell signalling components involved in cancer

**DOI:** 10.1186/1471-2407-5-125

**Published:** 2005-10-03

**Authors:** Michael O'Grady, Debasish Raha, Bonnie J Hanson, Michaeline Bunting, George T Hanson

**Affiliations:** 1 Invitrogen Corporation, 501 Charmany Drive, Madison, WI 53719 USA; 2 Invitrogen Corporation, 1600 Faraday Avenue, Carlsbad, CA 92008 USA

## Abstract

**Background:**

The transcription factor activator protein-1 (AP-1) has been implicated in a large variety of biological processes including oncogenic transformation. The tyrosine kinases of the epidermal growth factor receptor (EGFR) constitute the beginning of one signal transduction cascade leading to AP-1 activation and are known to control cell proliferation and differentiation. Drug discovery efforts targeting this receptor and other pathway components have centred on monoclonal antibodies and small molecule inhibitors. Resistance to such inhibitors has already been observed, guiding the prediction of their use in combination therapies with other targeted agents such as RNA interference (RNAi). This study examines the use of RNAi and kinase inhibitors for qualification of components involved in the EGFR/AP-1 pathway of ME180 cells, and their inhibitory effects when evaluated individually or in tandem against multiple components of this important disease-related pathway.

**Methods:**

AP-1 activation was assessed using an ME180 cell line stably transfected with a beta-lactamase reporter gene under the control of AP-1 response element following epidermal growth factor (EGF) stimulation. Immunocytochemistry allowed for further quantification of small molecule inhibition on a cellular protein level. RNAi and RT-qPCR experiments were performed to assess the amount of knockdown on an mRNA level, and immunocytochemistry was used to reveal cellular protein levels for the targeted pathway components.

**Results:**

Increased potency of kinase inhibitors was shown by combining RNAi directed towards EGFR and small molecule inhibitors acting at proximal or distal points in the pathway. After cellular stimulation with EGF and analysis at the level of AP-1 activation using a β-lactamase reporter gene, a 10–12 fold shift or 2.5–3 fold shift toward greater potency in the IC_50 _was observed for EGFR and MEK-1 inhibitors, respectively, in the presence of RNAi targeting EGFR.

**Conclusion:**

EGFR pathway components were qualified as targets for inhibition of AP-1 activation using RNAi and small molecule inhibitors. The combination of these two targeted agents was shown to increase the efficacy of EGFR and MEK-1 kinase inhibitors, leading to possible implications for overcoming or preventing drug resistance, lowering effective drug doses, and providing new strategies for interrogating cellular signalling pathways.

## Background

Cellular processes such as proliferation, differentiation, and death are regulated by signal transduction pathways which commonly exert their function through receptor mediated activation. The discovery in 1978 that the v-Src oncogene was a protein kinase led to a "cascade" of research into the role of kinases in cell-signalling pathways, and the subsequent finding that human cancer can result from the activity of nonviral, endogenous oncogenes, a major portion of which code for protein tyrosine kinases (PTKs) [1,2]. The epidermal growth factor receptor (EGFR) is a tyrosine kinase which acts as a master switch leading to activation of the transcription factor, activator protein-1 (AP-1), and other related pathways. The receptor itself is composed of extracellular, transmembrane, and tyrosine kinase domains. Ligand binding elicits a conformational change of the extracellular domain leading to receptor dimerization and subsequent transphosphorylation of intracellular domain tyrosines. The phosphorylated tyrosines act as binding sites for signal transducers initiating a series of kinase actions resulting in cellular proliferation and differentiation [3-5]. Aberrant signalling occurring from EGFR results in its conversion into an oncoprotein, and the consequent malfunction of cellular signalling networks leads to the development of cancers and other proliferative diseases. EGFR and its ligands are involved in over 70% of all cancers [[4,6], and [7]].

Hidaki, et.al. in the early 1980's discovered the first protein-kinase inhibitors, and established the principle of changing chemical structure to elicit different kinase inhibition specificity [8]. Drug development has followed the lead of the academic community in developing novel inhibitory compounds at points along these disease-related pathways. The protein kinase target class is now the second largest group of drug targets behind G-protein-coupled-receptors [3]. Kinases of the Tyrosine and Serine/Threonine family have been targeted successfully by small-molecule inhibitors and monoclonal antibodies, with many undergoing human clinical trials or successfully launched as therapeutic entities [9-13].

Acquired resistance to kinase-targeted anticancer therapy has been documented, and most extensively studied with imatinib (Gleevec™), an inhibitor of the aberrant BCR-ABL kinase, in chronic myelogenous leukemia [14]. Resistance has also occurred in EGFR-targeted inhibitor therapy using gefitinib (Iressa™) and erlotinib (Tarceva™). Mutations occurring in the catalytic domain of the receptor have been implicated in this resistance, but cannot account for all resistance seen to these small molecule inhibitors, indicating other mechanisms are involved in the resistance seen to date [15,16]. Therefore, multiple strategies will be necessary to overcome the observed resistance to these new molecularly targeted therapies, as well as methods to predict their efficacy.

Most kinase inhibitors target the ATP-binding site common to all kinases, and can bind multiple kinases [17]. This generates an inability to predict compound specificity for a particular kinase, and the subsequent need to analyze large numbers of kinases through a screening or profiling approach. Data from these *in vitro *assays allow the researcher to predict clinical uses for inhibitors and possible offsite target effects. Studies using purified kinase and substrate are dependent on ATP concentration used, and the apparent K_m _for ATP can differ between kinases. This can lead to problems in the development of small molecule inhibitors based on competition at the ATP-binding site of a kinase, as the ATP concentration *in vivo *may differ greatly from that used *in vitro*. In addition, kinase activity studies in a purified setting may use domains of proteins and peptide substrates, which can lead to erroneous interpretation of the true nature of kinase activity and/or inhibition. The *in vivo *studies using Western blot analysis also can be difficult to interpret due to the need to use a protein preparation from a cellular lysate, and inherent variability when using antibodies for Western blot analysis. Small changes in any step of the protocol could lead to differences in interpretation of the results. For these reasons, and the need for strategies to prevent or overcome resistance formation in malignancies, we have used an *in vitro *and functional cellular assay approach to study the EGFR/AP-1 signal transduction pathway. AP-1 activation through EGFR was assessed using a β-lactamase reporter gene assay, and served as a model for inhibition of pathway components on a functional cellular level. Kinase profiling using full length EGFR and peptide substrates was used in parallel for confirmation and specificity of inhibition. The knockdown of qualified targets in the EGFR/AP-1 pathway was further studied by immunocytochemistry, allowing for assessment on a cellular protein level. Following qualification of targets in this cancer-related pathway, a functional cellular assay was used to analyze the potential therapeutic benefit of combining RNAi and kinase inhibitors against EGFR and MEK-1 in the AP-1 activation pathway of a human cervical cancer cell line.

## Methods

### AP-1-bla ME180 CellSensor™cell line

An AP-1 response element (TGACTAA, 7X) was inserted into the MCS of the β-lactamase lentiviral reporter vector using Gateway^® ^technology according to the manufacturer's instructions (Invitrogen, Carlsbad, CA). The virus created from this vector was used to transduce ME180 cells [18] according to manufacturer's instructions (Invitrogen, Carlsbad, CA). Following a one day incubation, the cells were split and placed in media with Blasticidin (5 ug/ml) for 3–4 days. Cells were then washed with phosphate buffered saline (PBS), refed, and further selected for seven to ten days in media containing Blasticidin. Flow cytometry was used to select for β-lactamase expressing cells according to previously published protocols [19].

### EGF ligand stimulation of AP-1-bla ME180

AP-1-*bla *ME180 cells were plated in 96 well plates at a density of 20,000–25,000 cells/well in DMEM with 1 mM sodium pyruvate, 25 mM HEPES, 0.1 mM non-essential amino acids (Invitrogen Gibco, Grand Island, NY) plus 1% fetal bovine serum (FBS) and incubated overnight at 37°C with 5% CO_2_. The following day rhEGF (Calbiochem, San Diego, CA) was diluted in media with 1% FBS at desired concentrations, and cells incubated at 37°C with 5% CO_2 _for 5 hours. EGF treatment was performed at n = 8.

### β-lactamase reporter analysis

β-lactamase reporter gene expression was determined using the LiveBLAzer™ FRET B/G assay kit (Invitrogen Drug Discovery, Madison, WI) according to manufacturer's instructions.

### Inhibitor treatment of AP-1-bla ME180

Small molecule inhibitors (Calbiochem, San Diego, CA) were diluted in cell culture media at the desired concentration and preincubated with the cells for 30 minutes at 37°C with 5% CO_2_. EGF ligand stimulation was performed as indicated above followed by β-lactamase reporter analysis. DMSO was used as the negative control, per its use as the compound reconstitution medium. Final DMSO concentration in the medium was 0.05% for both compounds and negative controls.

### RNAi design and transfection of AP-1-bla ME180

dsRNAi Stealth™ oligos were designed against EGFR. The following sequences were used for the oligos:

EGFR: Sense 5' UUA GAU AAG ACU GCU AAG GCA UAG G 3'

Anti-Sense 5' CCU AUG CCU UAG CAG UCU UAU CUA A 3'

AP-1-*bla *ME180 cells were plated at a concentration of 10,000 cells/well in a 96 well plate and incubated overnight at 37°C with 5% CO_2_. AP-1-*bla *ME180 cells were transfected with 50 nM dsRNAi Stealth™ oligos and 2 μg/ml Lipofectamine 2000 according to manufacturer's suggestions (Invitrogen, Carlsbad, CA). mRNA was extracted from transfected cells 24 hours post transfection and EGFR expression levels determined by RT-qPCR using light upon extension (LUX™) primer sets (Invitrogen, Carlsbad, CA) for the target of interest and cyclophilin as a normalization control. Percent knockdown of the targeted message was determined as a ratio of target versus cyclophilin control.

Functional cellular analysis of the effect of RNAi knockdown was studied using the AP-1-*bla *ME180 CellSensor. The cells were transfected with ds RNAi oligos as above and incubated for various lengths of time prior to EGF stimulation for 5 hours and subsequent quantitation of β-lactamase using the LiveBLAzer FRET B/G assay kit (Invitrogen).

### Immunocytochemistry for cellular protein knockdown and phosphorylation status of EGFR

AP-1-*bla *ME180 cells were analyzed for EGFR knockdown at a cellular protein level following RNAi treatment. Cells were transfected with dsRNAi oligos as in the functional cellular studies above and incubated for ~60 hrs. The cells were then fixed with 4% Paraformaldehyde for 30 minutes at 22°C, followed by permeabilization in PBS + 0.25% Triton X-100 for 5 mins at 22°C. Blocking of antigen binding sites was performed with PBS + 2% FBS for 2 hours at 22°C with rocking. Cells were washed twice with PBS + 2% FBS, then incubated with rabbit anti-phospho-EGFR (Tyr1086) or mouse anti-EGFR (31G7) primary antibody (Invitrogen Zymed, San Francisco, CA) at 1:100 concentration and 1:25 concentration respectively in PBS + 1% FBS for 1 hour at 22°C with rocking. Following primary antibody incubation, the cells were washed twice with PBS + 1% FBS at 22°C with rocking. The secondary antibodies goat anti-rabbit IgG (H+L) Alexa Fluor^® ^488 and goat anti-mouse IgG (H+L) Alexa Fluor^® ^594 (Invitrogen Molecular Probes, Eugene, OR) were used at 1 μg/mL concentration in PBS + 1% FBS, and incubated at 22°C with rocking. Cells were washed three times with PBS + 1% FBS followed by addition of PBS + 10% glycerol for assay and storage. A Zeiss Axiovert 25CFL microscope with a FITC filter set (excitation D480/30X, emission D535/40 M, Chroma Technology Corporation, Rockingham VT) for Alexa 488, and a Texas Red filter set (excitation D560/40X, emission D630/60 M, Chroma Technology Corporation, Rockingham VT) for Alexa 594 was used for imaging of cells. Quantitation of knockdown was done using a Tecan Safire^® ^instrument using excitation wavelength of 485/7.5 nm and emission wavelength of 510/7.5 nm for the Alexa 488 labelled secondary antibody and excitation wavelength of 590/7.5 nm and emission wavelength of 615/7.5 nm for the Alexa 594 labelled secondary antibody.

### Immunocytochemistry for EGFR phosphorylation following kinase inhibitor treatment

AP-1-*bla *ME180 cells were treated with kinase inhibitors as outlined above. Immunocytochemistry using appropriate phospho-specific antibodies was performed as in the RNAi experiments to analyze EGFR autophosphorylation.

### Combining RNAi and kinase inhibitors on the AP-1-bla ME180 CellSensor

AP-1-*bla *ME180 cells were treated with 50 nM RNAi against EGFR as in experiments outlined above. Only one-half of a 96 well plate was treated, while the other half was mock treated with Lipofectamine™ 2000 (Invitrogen, Carlsbad, CA) alone. At 60 hours the cells were treated with a dilution series of AG1478 or U0126 as outlined above. Cells were then stimulated with EGF and the resulting β-lactamase readout was analyzed using GraphPad Prism^® ^to determine IC_50 _values. Controls for RNAi transfection (Med GC; 40–50% GC nucleotide content), EGFR knockdown (EGFR RNAi), and β-Lactamase knockdown (β-Lac) were used to set standards for transfection, single treatment, and maximal effects respectively.

### Kinase profiling of compounds used in functional cellular assays

Small molecule compounds used in the cellular assays were profiled in the SelectScreen™ Kinase Profiling Service (Invitrogen Drug Discovery Solutions, Madison, WI). This service utilizes the Z'-Lyte technology assay platform [21] where the biochemical assays were performed at a final concentration of 1 μM compound in 0.1% DMSO and an ATP concentration of K_m, app _for the EGFR protein. The assays were analyzed on a Tecan Safire^2^^® ^detection instrument. The percent inhibition values were calculated using XL fit 4.0 by ID-BS.

## Results

### ME180 EGFR/AP-1 signal transduction pathway is responsive to EGF stimulation

**Figure 1:**
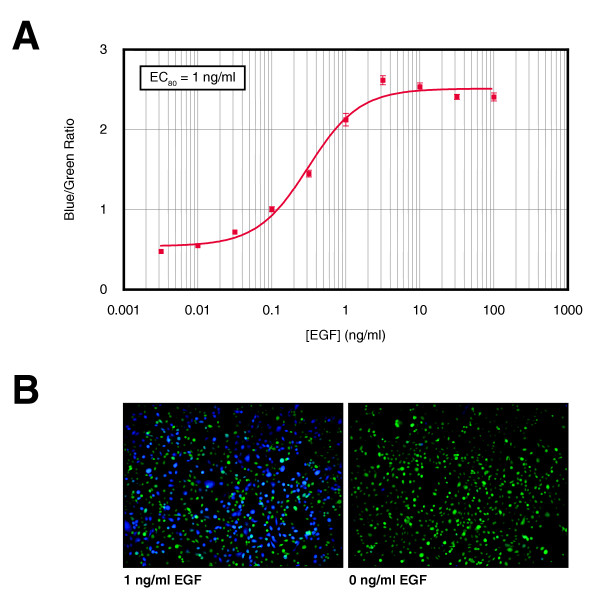
**EGF mediated activation in ME180AP-1 CellSensor**. EGF effectively stimulated the EGFR/AP-1 pathway in ME180 cervical cancer cells. Cells expressing β-lactamase were detected using a fluorescent plate reader (Panel A) or under fluorescent microscopy (Panel B). β-lactamase was measured using Invitrogen's LiveBLAzer™ FRET B/G assay. Ratios represent the mean of (± SEM) eight independent data points (n = 8).

In order to use the AP-1-*bla *ME180 CellSensor as a model system for combining targeted agents against components of a cancer-related pathway, we determined the dose-dependent response of the pathway to EGF stimulation. The EC_50 _was determined to be 0.31 ng/mL from the blue:green ratiometric readout of the β-lactamase assay (Figure 1A). Imaging capabilities made possible with the β-lactamase reporter system allow for microscopic visualization of reporter enzyme activity due to cleavage of the FRET-based substrate (Figure 1B). To maximize the assay window while maintaining sensitivity to inhibition by putative inhibitors, an EC_80 _concentration of 1 ng/ml EGF was used to stimulate pathway response in inhibition assays.

### EGFR/AP-1 signal transduction can be inhibited at multiple points in the pathway

The commercial availability of known small molecule kinase inhibitors allowed for the study of their potency in our cellular system. Prior to analyzing efficacy in a functional cellular assay, we tested their inhibition of EGFR in a biochemical assay using Z'-LYTE, a FRET-based platform.

**Figure 2:**
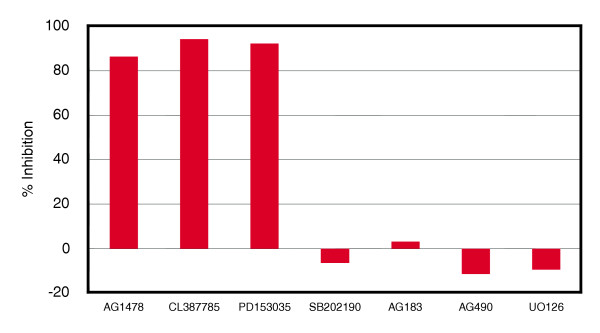
**Biochemical assay results with kinase inhibitors**. Percent inhibition values determined by using the Z'-LYTE technology platform (Invitrogen), and single point concentration testing (1 μM) with two independent data points (n = 2). Small molecule compounds CL387785, PD153035, and AG1478 show strong inhibition of the EGFR, while others tested exhibit none.

The small molecule compounds are structurally related with some targeting the ATP binding site of the kinase (AG1478, CL387785, PD153035, SB202190) [20], and others targeting the tyrosine substrate binding site (AG490, AG183) [4,22]. U0126 acts in the unique manner of blocking activation of MEK through a mechanism independent of the ATP binding site [12,23]. The kinase inhibitors tested show a spectrum of inhibition against EGFR (Figure 2), and correlate well with published results of inhibitor specificity [17].

**Figure 3:**
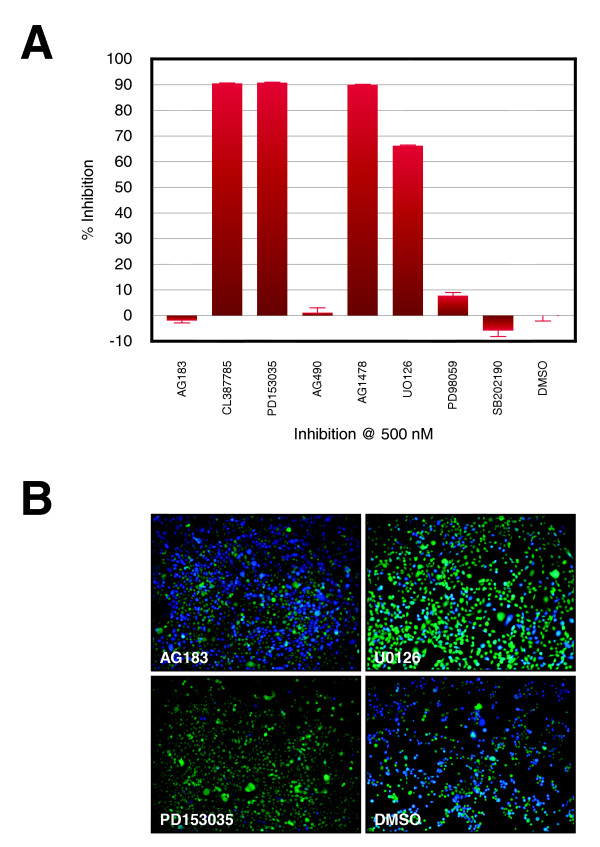
**Kinase inhibitors block activation at multiple points in EGFR/AP-1 pathway**. Inhibitors decrease the response of the AP-1-*bla *ME180 CellSensor when stimulated with EGF. β-lactamase was measured using Invitrogen's LiveBLAzer™ assay (Panel A). Percent inhibition is determined in comparison to untreated sample and represents the mean of (± SEM) eight independent data points (n = 8). Photomicrographs of cells were obtained under fluorescent microscopy (Panel B).

The inhibitors were next tested for their effectiveness in inhibiting the EGFR/AP-1 pathway in a functional cellular assay. Initial analysis was performed with an inhibitor concentration of 500 nM. Results indicate the CL387785, PD153035, and AG1478 compounds strongly inhibit AP-1 gene activation (Figure 3A), indicating that targeting of EGFR leads to a potent reduction in AP-1 gene expression in ME180 EGF stimulated cells. AG183 shows no inhibition at 500 nM and also showed no inhibition of EGFR in the biochemical assay (Figure 2). The U0126 compound, which targets MEK-1, also shows inhibition of the EGFR/AP-1 pathway (Figure 3A), an expected result when targeting another component of the EGFR/AP-1 pathway. The p38 MAP kinase inhibitor SB202190 shows no inhibition of the pathway in the AP-1-*bla *ME180 CellSensor as could be predicted based on known pathway components. The live cell assay format allows for imaging and further confirmation of the inhibition shown in the quantitative panel (Figure 3B).

**Figure 4:**
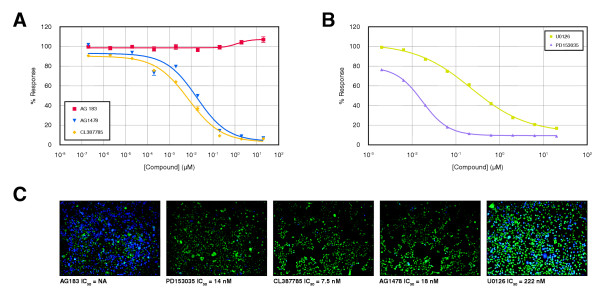
**Potency of individual inhibitors towards kinases in the AP-1 pathway determined in a dose response manner**. Inhibitors were used in a dose response manner on the AP-1-*bla *ME180 CellSensor, and β-lactamase measured using Invitrogen's liveBLAzer™ FRET B/G assay (Panels A and B). IC_50 _determinations were performed using GraphPad Prism^® ^and values shown with images of inhibitors at 0.2 μM concentration (Panel B).

The inhibitors that tested positive for inhibition in the functional cellular assay at a 500 nM concentration were further studied in a dose response manner. Inhibitor potency could be assigned through IC_50 _values determined on a cellular pathway level. The EGFR inhibitors CL387785, PD153035, and AG1478 show increased efficacy of pathway inhibition when compared to U0126, as might be expected when targeting the initial component of this receptor linked pathway (Figure 4A, B, and 4C).

### Cellular phosphorylation of EGFR on a cellular protein level matches reporter gene results

**Figure 5:**
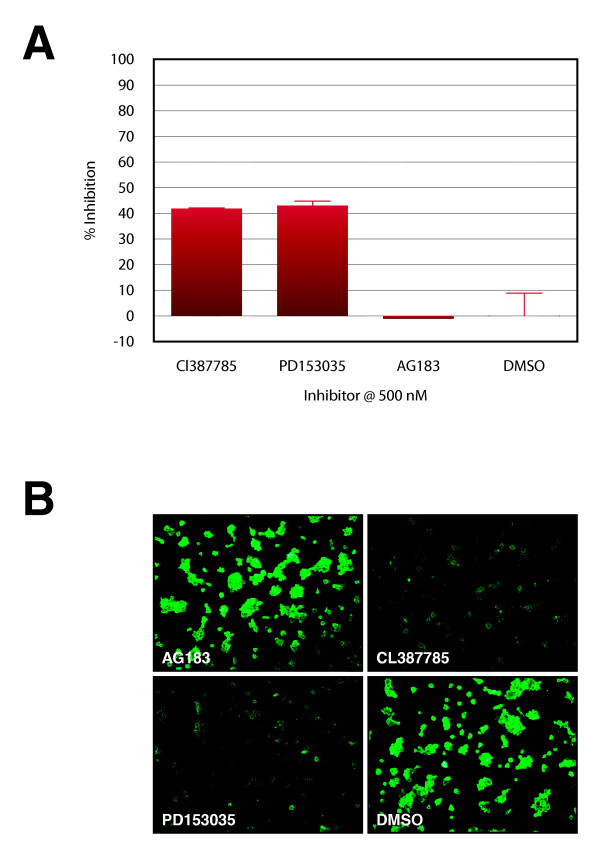
**Autophosphorylation of EGFR shows similar inhibition profile as the AP-1-*bla *ME180 functional cellular assay**. Immunocytochemistry with phospho-specific antibodies demonstrates the same inhibition profile as the AP-1 activation assay performed using the ME180 CellSensor. Fluorescence was measured from the Alexa fluor 488 and 594 labelled secondary antibodies used for detection (Panel A). Percent inhibition was determined in comparison to untreated sample and represents the mean of (± SEM) eight independent data points (n = 8). Images were obtained from the Alexa fluor 488 labelled secondary antibody under fluorescent microscopy (Panel B).

Confirmation on a cellular protein level of the results obtained using kinase inhibitors on the EGFR/AP-1 pathway in a cell-based reporter readout was obtained through immunocytochemistry. Inhibition of autophosphorylation was shown by using antibodies specific for the phosphorylated tyrosine-1086 of EGFR. PD153035 showed strong inhibition of EGFR autophosporylation, while AG183 did not (Figure 5A and 5B) validating the results seen in the functional cellular assay (figure 4).

### RNAi qualifies EGFR as a target for AP-1 pathway inhibition

**Figure 6:**
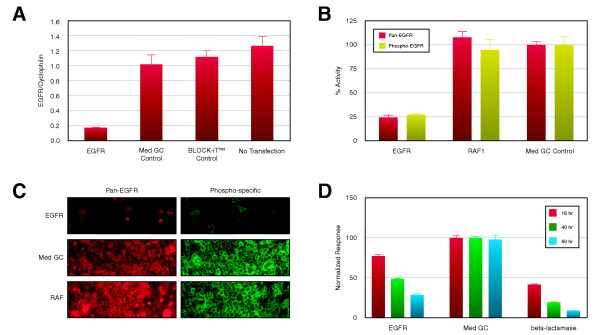
**Knockdown by RNAi qualifies EGFR involvement in ME180 AP-1 CellSensor pathway**. AP-1-*bla *ME180 cells transfected with RNAi targeting EGFR were analyzed by RT-qPCR (Panel A). EGFR mRNA expression is knocked down ~80% as compared to a non-specific control (Med GC). Results are represented as a ratio using cyclophilin as a control. Cells RNAi treated with oligos specific for EGFR show a reduced level of EGFR protein on the cell surface including a loss of autophosphorylation (Panels B and C). RFU represent measurement of the Alexa fluor 488 and 594 labelled secondary antibodies used for detection of the phospho and pan EGFR antibodies and represent the mean of (± SEM) eight independent data points (n = 8). RNAi treated AP-1-*bla *ME180 cells also exhibit a knockdown of AP-1 activation when EGF stimulated and analyzed at different timepoints (Panel D). Normalized response represents the amount of AP-1 gene activation present when compared with a non-specific control (Med GC), and represent the mean of (± SEM) eight independent data points (n = 8).

dsRNAi towards EGFR was shown to knockdown the total amount of EGFR present as well as the autophosphorylation of the receptor. Analysis of knockdown was analyzed by RT-PCR demonstrating an ~80% knockdown on an mRNA level (Figure 6A). Immunocytochemistry performed on RNAi treated AP-1-*bla *ME180 cells shows a knockdown of EGFR on a cellular protein level with a concomitant loss of autophosphorylation of the receptor (Figure 6B and 6C). Analysis of the effect of RNAi knockdown in a functional cellular assay confirmed the essential role of EGFR in the activation of AP-1 (Figure 6D). In addition, lower receptor levels result in less pathway response, and the stronger effect on AP-1 activation correlates with RNAi incubation time. Additional controls using a low GC (30–40% GC content) dsRNAi oligo and dsRNAi oligos targeting genes not involved in the EGFR/AP-1 pathway (NF-κB, IKK-α) show no effect on a cellular protein level or in a functional cellular assay (data not shown).

### Combining EGFR RNAi with AG1478 or U0126 increases the potency of the kinase inhibitors against AP-1 activation

**Figure 7:**
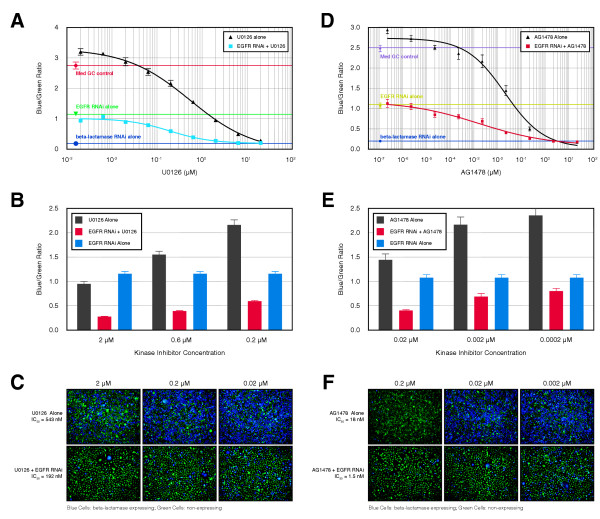
**Combining RNAi toward EGFR and the kinase inhibitors U0126 and AG1478 increases the potency of the small molecule compounds**. Small molecule kinase inhibitors shown to have inhibitory properties in the AP-1 CellSensor assay were used in tandem with RNAi. RNAi targeting the EGFR was kept constant while U0126 targeting MEK-1 kinase (Panel A) and AG1478 targeting EGFR (Panel C) were used in a dose response manner to examine potency effects of the combined treatment. The shift in IC_50 _indicates the "cocktail" effect seen with the tandem treatment.

To determine whether there were additive effects, which could be relevant in therapeutic efficacy and prevention of drug resistance (15,16, and 24), we performed experiments using small molecule inhibitors in combination with RNAi. U0126, a known MEK-1 inhibitor, exhibits an IC_50 _of 543 nM when used in isolation on the AP-1 cell line. Upon combination with RNAi against EGFR, the IC_50 _for the MEK-1 inhibitor shifts 2.5–3 fold more potent to 192 nM (Figure 7A, B, and 7C). AG1478, an EGFR inhibitor, exhibits an IC_50 _of 18 nM in isolation, but shifts to 1.5 nM when used in tandem with RNAi targeting EGFR (Figure 7D, E, and 7F). The results indicate the validity of using a functional cellular assay for the preclinical analysis of putative benefits when combining kinase inhibitors with other targeted agents of signal transduction pathways related to cancer.

## Discussion

Molecular targeted therapies against signalling pathway components involved in cancer have arrived in the clinic, and drug discovery efforts are increasing in this evolving area [[3,9,11,13,24], and [25]]. The growth of screening for small molecule compounds which act as kinase inhibitors has led to their becoming the second most targeted group of druggable entities after G-protein-coupled receptors [26].

Modulation of kinase activity can be accomplished by strategies other than inhibition of phosphorylation activity through the blocking of ATP binding. Such methods include disruption of protein-protein interactions and the knockdown or downregulation of kinase gene expression by antisense or RNA interference approaches [16,24]. The need for multiple approaches for therapies targeting kinases can be seen in the reports of resistance towards the recently launched kinase inhibitors gefitinib (Iressa™) and imatinib (Gleevec™), which inhibit the EGFR and Bcr-Abl kinase respectively [14,27]. Strategies implemented for overcoming or preventing this resistance have included chemical modifications of the inhibitor compound using a rational drug design strategy to increase the potency against the targeted kinase. The ATP binding site of kinases has proven to be a "hot spot" for kinase mutations and includes a "gatekeeper" region shown to be difficult to block with inhibitors and their new more potent derivatives. Recent work has been published on inhibitors targeting the substrate binding site of the Bcr-Abl kinase [28], which seem to inhibit wild-type and all imatinib-resistant kinase domain mutations, including the "gatekeeper" mutation [29]. These approaches have proven successful for imatinib and will undoubtedly work in the near term for other small molecule inhibitors, but experience suggests the possibility of further mutations leading to increased resistance [[15,16], and [24]].

Blockade of one kinase alone might not be sufficient to achieve needed pathway inhibition, and so targeting of multiple kinases could be more promising in terms of efficacy and prevention of resistance. This type of combinatorial therapeutic approach has become the standard for HIV treatment to maximize potency, minimize toxicity, and diminish the risk for resistance development [30]. Targeted compounds could be used together or in combination with toxic agents such as chemotherapy or ionizing radiation, as well as with other novel agents. This approach has proven successful in recent studies using chemotherapeutic agents or ionizing radiation in combination with kinase inhibitors [[31-33] and [34]]. However, given the rapidly growing number of inhibitory agents and an exponential number of possible combinations, it will not be possible to test all such groupings in a clinical trial setting [16,24]. The need for predictive preclinical models allowing for the choice of which studies to advance through the drug discovery process led to the experiments outlined in this paper with RNAi and the kinase inhibitors U0126 and AG1478.

First, in order to establish a functional cellular model for investigation of the EGFR/AP-1 pathway we built a stable cell line responsive to EGF stimulation. Kinase inhibitors and RNAi analysis established components that were involved in the functional response of the EGFR/AP-1 pathway that could be inhibited by molecularly targeted agents.

Biochemical analysis of EGFR inhibition using the same small molecule inhibitors further supported our cellular results and demonstrated the importance of complimentary approaches when analyzing signal transduction pathways. Inhibitors such as U0126 inhibit the functional response of the AP-1 pathway as shown in the cellular reporter readout, but work at a level independent of EGFR inhibition determined by *in vitro *assays.

RNAi analysis and immunocytochemistry further demonstrated the essential role EGFR plays at the beginning of the AP-1 activation cascade. Showing that inhibition or knockdown of the receptor leads to the functional results observed in the cellular assay model, led us to analyze combinatorial effects of using RNAi and kinase inhibitors in tandem. When the targeted agents were used together, an increased potency of the inhibitors was observed. This finding demonstrates the use of a cellular reporter system to predict the cocktail effects of a growing number of targeted agents against cell signalling components involved in cancer.

## Conclusion

Our results demonstrate the essential role for EGFR in AP-1 activation when analyzed using an ME180 cervical cancer cell line. Confirmation of "druggable" targets was shown using the parallel approach of known kinase inhibitors and RNA interference. An immunocytochemistry approach using phospho- and pan-specific EGFR antibodies strengthened the argument for the use of the AP-1-*bla *ME180 cell line as a viable model for the analysis of a combinatorial targeted agent approach to cancer-related pathways. Results obtained combining RNAi toward EGFR and small molecule inhibitors of MEK-1 and EGFR indicate a beneficial effect on EGFR/AP-1 pathway inhibition. This cellular model approach could lead to further studies combining kinase inhibitors with other targeted agents, and suggests possible implications for screening of compound libraries to uncover novel pathway inhibitors.

## Abbreviations

AP-1 = Activator Protein 1, *bla *= beta-lactamase, EGFR = Epidermal Growth Factor Receptor, MEK1 = Mitogen Activated Protein Kinase Kinase 1, Phosphate Buffered Saline PBS, PTKs = Protein Tyrosine Kinases, RNAi = RNA interference.

## Competing interests

The authors are all employees of Invitrogen Corporation whose products are used in this article for research purposes.

## Authors' contributions

MO performed all experiments involving the ME180 AP-1 CellSensor with kinase inhibitors, RNAi, and immunocytochemistry, as well as coordinating and executing the studies performed. BJH assisted with immunocytochemistry as well as coordination and execution of the study. MB and DR assisted and coordinated RNAi experiments included in the study. GTH assisted in all experiments, as well as in the coordination and execution of the study. All authors read and approved the final manuscript.

## Pre-publication history

The pre-publication history for this paper can be accessed here:



## Supplementary Material

Additional File 1 **EGFR/AP-1 pathway **Schematic representation of EGFR/AP-1 pathway indicating components targeted by inhibitors or RNAi. Click here for file
