# Supplementary figures and images for: Combining RNA interference and kinase inhibitors against cell signalling components involved in cancer
Source: BMC Cancer. 2005 Oct 3;5:125. doi: 10.1186/1471-2407-5-125 (PMC1262698; doi:10.1186/1471-2407-5-125)

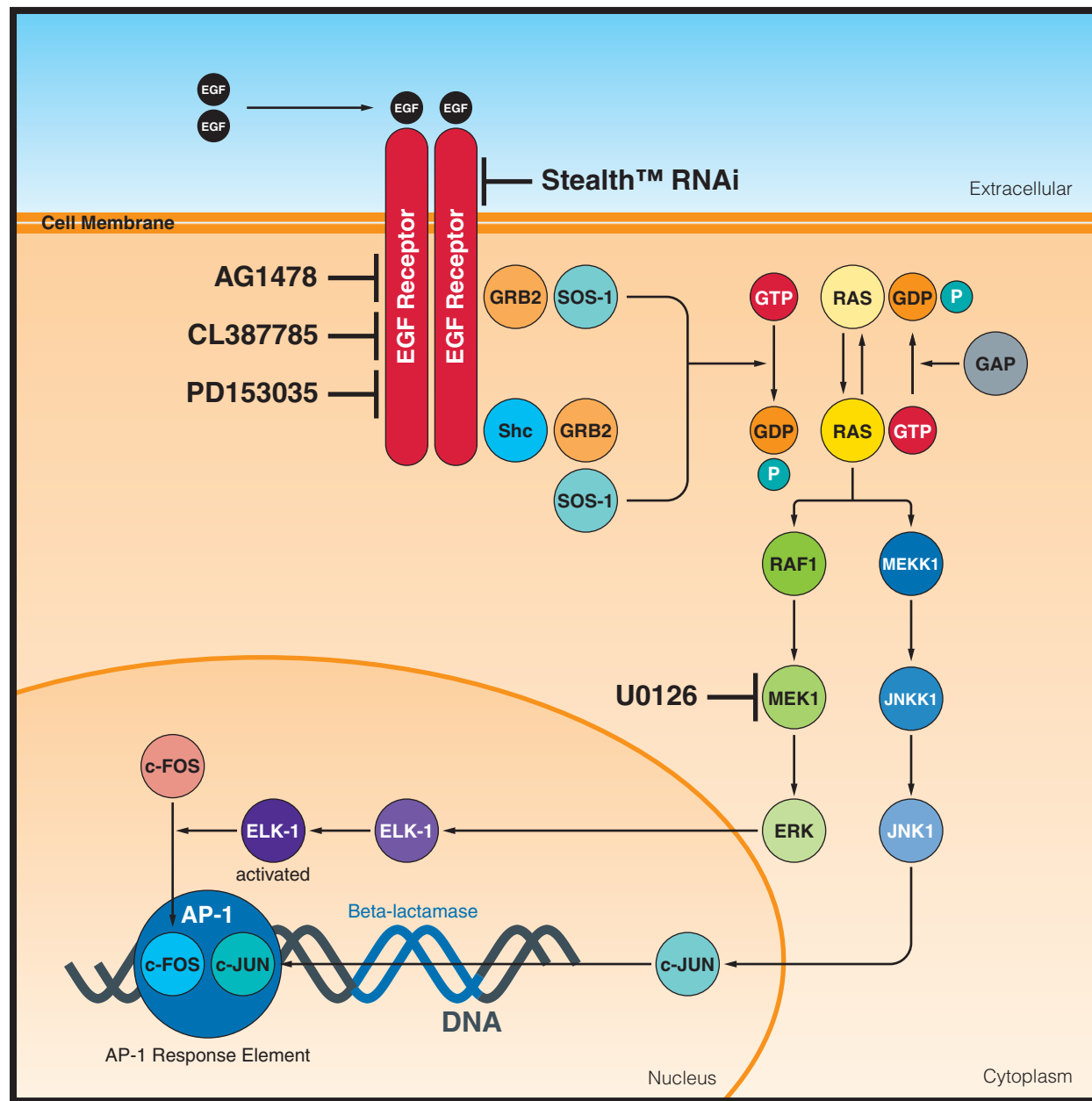

Supplement: Additional File 1 — EGFR/AP-1 pathway Schematic representation of EGFR/AP-1 pathway indicating components targeted by inhibitors or RNAi. [file 1471-2407-5-125-S1.pdf]
